# How accurate are modelled birth and pregnancy estimates? Comparison of four models using high resolution maternal health census data in southern Mozambique

**DOI:** 10.1136/bmjgh-2018-000894

**Published:** 2019-07-01

**Authors:** Yolisa Prudence Dube, Corrine Warren Ruktanonchai, Charfudin Sacoor, Andrew J Tatem, Khatia Munguambe, Helena Boene, Faustino Carlos Vilanculo, Esperanca Sevene, Zoe Matthews, Peter von Dadelszen, Prestige Tatenda Makanga

**Affiliations:** 1 Faculty of Science and Technology, Surveying and Geomatics, Midlands State University, Gweru, Zimbabwe; 2 Department of Geography and Environment, University of Southampton, Southampton, UK; 3 Centro de Investigacao em Saude de Manhica, Manhica, Mozambique; 4 Department of Geography and Environment, University of Southampton, Southampton, UK; 5 Flowminder Foundation, Stockholm, Sweden; 6 Department of Social Statistics and Demography, University of Southampton, Southampton, UK; 7 Department of Women's Health, King's College London, London, UK

**Keywords:** health geography, demographic distribution, comparison, maternal health census, live births and pregnancies

## Abstract

**Background:**

Existence of inequalities in quality and access to healthcare services at subnational levels has been identified despite a decline in maternal and perinatal mortality rates at national levels, leading to the need to investigate such conditions using geographical analysis. The need to assess the accuracy of global demographic distribution datasets at all subnational levels arises from the current emphasis on subnational monitoring of maternal and perinatal health progress, by the new targets stated in the Sustainable Development Goals.

**Methods:**

The analysis involved comparison of four models generated using Worldpop methods, incorporating region-specific input data, as measured through the Community Level Intervention for Pre-eclampsia (CLIP) project. Normalised root mean square error was used to determine and compare the models’ prediction errors at different administrative unit levels.

**Results:**

The models’ prediction errors are lower at higher administrative unit levels. All datasets showed the same pattern for both the live birth and pregnancy estimates. The effect of improving spatial resolution and accuracy of input data was more prominent at higher administrative unit levels.

**Conclusion:**

The validation successfully highlighted the impact of spatial resolution and accuracy of maternal and perinatal health data in modelling estimates of pregnancies and live births. There is a need for more data collection techniques that conduct comprehensive censuses like the CLIP project. It is also imperative for such projects to take advantage of the power of mapping tools at their disposal to fill the gaps in the availability of datasets for populated areas.

**Key questions**

**What is already known?**

- It is fundamental to accurately identify populations at risk by unmasking the heterogeneities that exist at very high spatial resolutions.
- There is need to validate the performance of global demographic distribution models and continue improving their performance especially at high spatial resolutions.

**What are the new findings?**

- Geocoded health data can be used as input data to improve the estimation power of demographic distribution datasets and to validate them.
- The quantified impact of spatial resolution and accuracy of input data on the performance of the models revealed the importance of high spatial resolution health data.

**What do the new findings imply?**

- This study shows the significance of incorporating geocoded data and geographical methods in clinical research as they add value to modelling demographic distribution estimates for maternal health.

## Introduction

The key to promoting universal health coverage is to expose any hidden gaps in health service provision using sufficiently disaggregated geographical data that is reliable.^1^ Thematic mapping, spatial analysis and spatial modelling have been identified as the Geographical Information Systems (GIS) methods that are valuable in policy discussions pertaining to maternal and perinatal health, relying greatly on volume, completeness, timeliness and accuracy of data.^2^ In many low-income and middle-income countries (LMIC), which contribute 99%,^3^ of the 830 women who die every day around the world due to pregnancy and child birth complications with half of these deaths occurring in sub-Saharan Africa,^4 5^ data on maternal and perinatal distributions are not routinely or accurately collected. Their national level estimates are mostly only available from censuses that are conducted after 10-year timelines at best.^6^ Considering the significance of GIS methods and data in measuring progress in improving maternal and perinatal health and formulating relevant policies, new methods have been developed to generate these data and make them widely available to end users.^3^ Global population and demographic distribution datasets such as Gridded Population of the World,^7^ Global Rural-Urban Mapping Project,^8^ LandScan^9^ and Worldpop^10 11^ (combination of AfriPop, AsiaPop and AmeriPop) have been developed to address issues of availability of such geographical data for LMICs. They include yearly estimates of population and demographic distributions. The Worldpop dataset is a widely used high resolution dataset, created to address the lack of demographical data in LMICs, which is used by 95% of the countries mapped by the project and international organisations, foundations and agencies including the WHO, The World Bank, Bill & Melinda Gates Foundation, Clinton Health Access Initiative and Red Cross International.^10^

The introduction of the Millennium Development Goals (MDG) prompted the extensive use of these global population and demographic distribution datasets, especially in low-income regions, to derive health metrics for applications in developing intervention programmes aimed at achieving these goals.^12^ The justification for their utilisation is that they are standardised and considered to be of acceptable accuracy for national scale applications.^13^ Such justification was acceptable since efforts made towards achieving the MDGs within the set deadline of 2015 focused on national level adjustments.^14 15^ Studies like Hay and others,^16^ Gething and others,^17^ Soares and Clements,^18^ Schur and others^19^ and so on have used these datasets at high spatial resolutions.^12^ Studies have validated the global datasets at subnational scale and revealed their level of accuracy at such scales, while recommending methods for improving the level of accuracy at subnational scales.^20 21^

Existence of inequalities in access to healthcare services and quality of healthcare at subnational levels has been identified despite decline in maternal and perinatal mortality rates at national levels, leading to the need to investigate such conditions using geographical analysis methods.^2^ The use of data at highest level of disaggregation, to avoid masking of existing heterogeneity, will produce a sincere depiction of the progress in maternal and perinatal healthcare in LMICs. Accurate geographical analyses at subnational levels are therefore of great necessity, requiring accurate geographical data. The need to assess accuracy of global population and demographic distribution datasets at all subnational levels arises from the current emphasis on subnational monitoring of maternal and perinatal health progress. This has been brought about by the new targets stated in the Sustainable Development Goals (SDGs) announced by the United Nations (UN) in the year 2016,^22^ which include the goal to reduce maternal mortality ratio to less than 70 per 100 000 live births by the year 2030.^5^

It is fundamental to accurately identify populations with the most need of healthcare interventions to effectively evaluate the performance of healthcare systems.^22^ This provides evidence to support decision making concerning (1) planning for safer births and healthier new-borns and (2) resource allocation and improving access to maternal and perinatal healthcare as this is one of the main focuses in healthcare delivery.^2 23^ Inaccurate identification of the populations in need of maternal healthcare interventions has been one of the causes of the variations in the utilisation of maternal healthcare.^24^ The use of poor information in research and policy making leads to inefficient allocation of limited resources deterring the desired achievement of improved maternal and perinatal health quality. A true representation of the maternal and perinatal population distribution is therefore crucial in the successful implementation of interventions and it can only be achieved using accurate and highly disaggregated geographical data. Emphasis is on accuracy and detail of the population distribution datasets as their applications have become more intensive and their implications more pronounced in the achievement of the new SDGs.^25^

The desire to perform analyses at higher spatial resolutions has brought about the need to use the available datasets at high levels of disaggregation. As a source of data that is widely used in data deficient regions, the Worldpop dataset creators are constantly improving the disaggregation methods to refine the dataset for use at high spatial resolutions.^26 27^ It is imperative therefore, whenever data are available, to validate the dataset’s level of accuracy at small spatial scales to inform of the performance of the methods used. With the limited resources, available for the healthcare intervention programme for the low-income regions, there is need for accurate input data for analyses done prior to making decisions to ensure targeting of the right population groups. Knowledge of the level of accuracy of the data they are using allows the end users to factor in uncertainty brought about by the degree of accuracy of their input data. The assessment of the datasets brings the aspect of reliability to the attention of the users, thus cultivating a culture of always considering uncertainty of the data. Quantifying the errors within the datasets encourages the users to also quantify the levels of uncertainties of the results obtained before decision making.

Currently, Worldpop datasets available for Mozambique include population at the 100 m scale for the years 2010 and 2015 as well as pregnancies and live births datasets at the 1 km scale for the year 2015. The gridded estimates of pregnancies and live births were created by integrating sources like UN statistics, household survey data, age-specific fertility data, growth rates, live births, still births and abortions and converting the women of reproductive age (WRA) dataset constructed from satellite derived maps of land cover and settlements.^28^ Methods outlining how the live births dataset is created are outlined elsewhere,.^29^ Accuracy of the datasets is broadly dependent on the availability and accuracy of the input data for a specific region, such as recent census data or Demographic and Health Surveys (DHS) data. Specifically, the output estimates of live births and pregnancies are dependent on the following:

1. Accuracy of the input population dataset (whose accuracy is dependent on the temporality and availability of country-specific data including census data, land cover data, night-time lights imagery, road networks and so on and the UN World Population Prospects and UN World Urbanisation Prospects estimates.).
2. Accuracy and availability of the region-specific age-specific fertility rates (ASFRs) and age structure data from data sources such as the DHS and UN population estimates.

The accuracy of input demographic census data is limited by errors due to consideration of persons as residents of more than one household, declaration of period and households and errors in mortality data due to possible dissolution of households due to death of members.^30^ The limited level of training of interviewers and questions in censuses is a cause for concern with census data quality, having led to the need for follow-up surveys.^31^ In the case of Mozambique, the indistinct definition of demographic indicators and relevant survey design are problems that are still being addressed.^31^ Such inherent sources of errors in census data introduce uncertainty in the accuracy of the input demographic census data.

Despite the importance of detailed and timely census data, less work has been done in enumerating actual live births and pregnancies over small spatial scales. The Community Level Intervention for Pre-eclampsia (CLIP) trial (ClinicalTrials.gov number ID NCT01911494) in Mozambique was a cluster randomised control trial, testing if a level package of care entailed early identification of women with high chances of experiencing pregnancy complications. Identifying women at risk was achieved through the use of community health workers equipped with mobile phone based point of care tools and decision aids.^32^ The baseline phase of the trial involved carrying out global positioning system (GPS) household surveys, where the information about all WRA in each household in the study area was captured. The information included the age of the woman, their pregnancy status and number of live births to the woman.^20^ The CLIP baseline data therefore represent a much more detailed and geographically precise input data source likely to improve modelled Worldpop births and pregnancies data, allowing for validation of estimates using known geotagged maternal and child data with high spatial and temporal resolutions. This research aims to quantify and assess the model improvement of estimated pregnancies and livebirths, using CLIP data enumerating actual live births and pregnancies for regions in the provinces of Gaza and Maputo in Mozambique. The objectives of this research were to:

- Estimate live births and pregnancies datasets for the Gaza and Maputo regions using the CLIP baseline data as an additional input data source for the Worldpop process.
- Quantify differences in model performance and error between the births and pregnancy estimates generated using the CLIP data vs standard input data sources.
- Quantify the resulting impact of the models on estimates of live births and pregnancies.

## Methods

### CLIP trial

**Figure 1:**
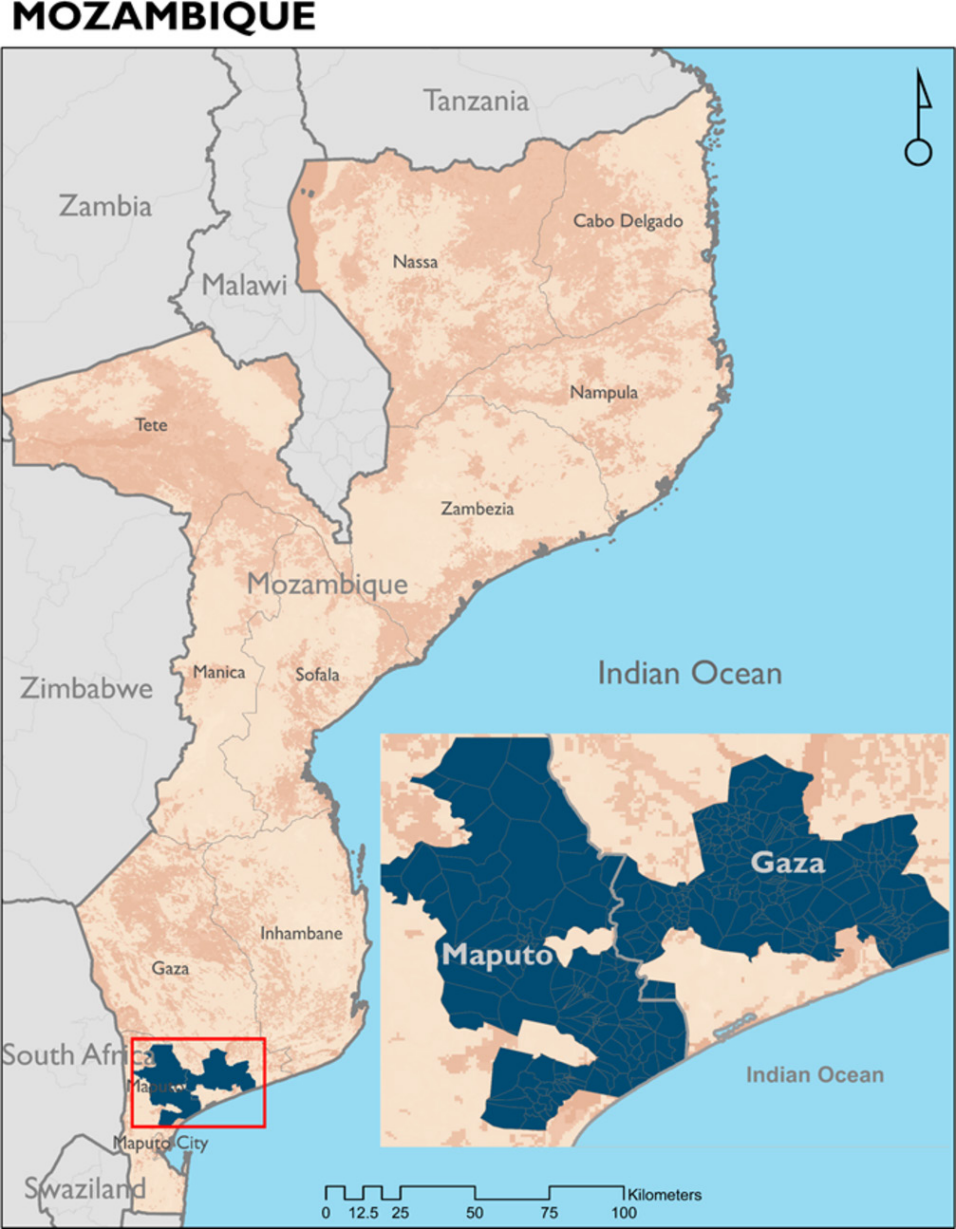
Study sites, Maputo and Gaza provinces in Southern Mozambique.

Figure 1 shows the study sites in southern Mozambique. Data were collected in parts of the two provinces of Gaza and Maputo. The administrative unit divisions shown in the insert are the neighbourhood units (referred to as admin 5 units in this paper). The CLIP study represents a household census of all households in 12 villages with WRA (12–49 years) conducted from March to October 2014 in Maputo and Gaza provinces of southern Mozambique. The regions had to contain a minimum population of 25 000 inhabitants that would result in at least one maternal death per year as per data from the 2007 national census.^33 34^ The inclusion criterion for the WRA was having lived in the household for more than 30 days prior to the date of the census and having the intention to live in the household as a permanent resident for at least 6 months following the census.^33^ A total of 50 493 households and 80 483 WRA (mean age 26.9 years) were surveyed. Admin 5 level data for age-specific number of WRA, pregnancies and live births and GPS coordinates of the households with WRA were collected as part of the baseline work for the CLIP trial.^33^ Admin 5 boundaries were generated by creating Thiessen polygons around GPS points with the same neighbourhood name. Higher level administrative boundaries (admins 4, 3, 2 and 1) were then derived from these lower level data and the corresponding age structure data (http://www.ine.gov.mz/estatisticas/estatisticas-demograficas-e-indicadores-sociais/populacao/relatorio-de-indicadores-distritais-2007) joined to each layer. To the authors’ knowledge, the CLIP data on pregnancies and live births is the most granular dataset there is in this region of Mozambique. We also anticipate that due to the rigorous attempts to identify all WRA, by visiting all households in the study area, the data are likely the most accurate representation of pregnancies and livebirths in the study area, hence the choice to use the data as part of data creation and comparison processes.

### Region-specific births and pregnancies model

Two models of live births and pregnancies were created, using admin 5 level data and the other using admin 3 level data. Births and pregnancy datasets were generated using Worldpop methods highlighted in James *et al*,^35^ with the addition of region-specific data as obtained through the CLIP project, including ASFRs, births-to-pregnancy ratios and number of births, pregnancies and WRA. Spreadsheets of ASFRs for admin 3 and admin 5 were generated by dividing age-specific births by age-specific WRA, while the pregnancy-to-birth multiplier was created for the study region by dividing the total number of pregnancies by total births for each admin 5 unit (and admin 3) and averaging the multipliers to get a value for the whole region. The Worldpop adjusted 2010–2015 population dataset^36^ was clipped to the extent of the study region and used in the generation of the age-specific WRA raster layers. These region-specific births and pregnancy datasets were created at varying spatial scales to determine the effect of input spatial resolution on model performance. To eliminate the error introduced by inaccurate census data, the births raster dataset was adjusted by multiplying it by the CLIP births raster at each admin 5. This step ensured the error in the adjusted births dataset would be due to disaggregation only.

$$Births=ASFR_{CLIP}\times WRA$$

WRA=proportionofwomen×agegroupproportion×population

The three datasets used to create the WRA dataset were created using census data, which as stated above, can be inaccurate. The ASFR dataset used is the CLIP dataset, hence the dataset that needs adjusting is the WRA dataset, which can be adjusted by adjusting the births dataset. Adjusting this dataset was a method used to eliminate the error due to inaccurate input census data. The adjustment factor was computed using the formula below:

$$Adjustmentfactor=\frac{Births_{CLIP}}{Births}=\frac{ASFR_{CLIP}\times WRA_{CLIP}}{ASFR_{CLIP}\times WRA}$$

The adjusted births dataset becomes:

Adjustedbirths=Births×Adjustmentfactor

**Figure 2:**
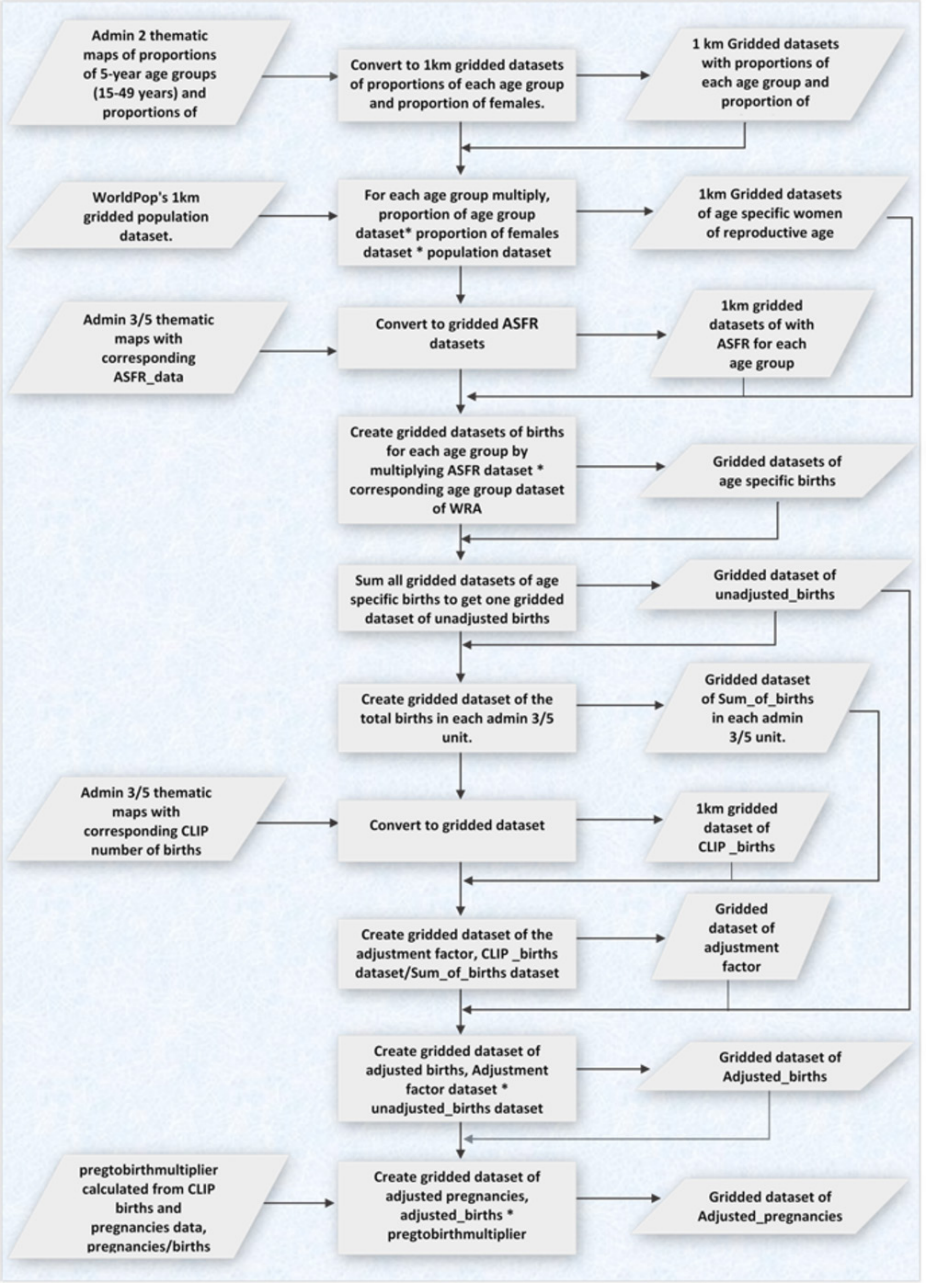
Data generation process for model comparison. CLIP, Community Level Intervention for Pre-eclampsia.

This was possible because the ASFR values used to create the dataset were computed from the CLIP data, meaning that adjusting the dataset using the number of births at each admin 5 unit resulted in adjusting the WRA computed using the age structure data and the Worldpop population dataset. This meant that the error in the resulting dataset was due to disaggregation. The process of recreating the datasets is shown in figure 2.

### Model comparison

The analysis involved comparison of four models: (1) CLIP model only (thematic maps with corresponding values for live births and pregnancies generated from the household survey); (2) admin 5 Worldpop-CLIP model (Worldpop methods incorporating region-specific input data at admin 5 level, as measured through the CLIP project); (3) admin 3 Worldpop-CLIP model (Worldpop methods incorporating region-specific input data at admin 3 level, as measured through the CLIP project) and (4) Worldpop-only model, using standardised input data as published through the Worldpop project.^29^

**Figure 3:**
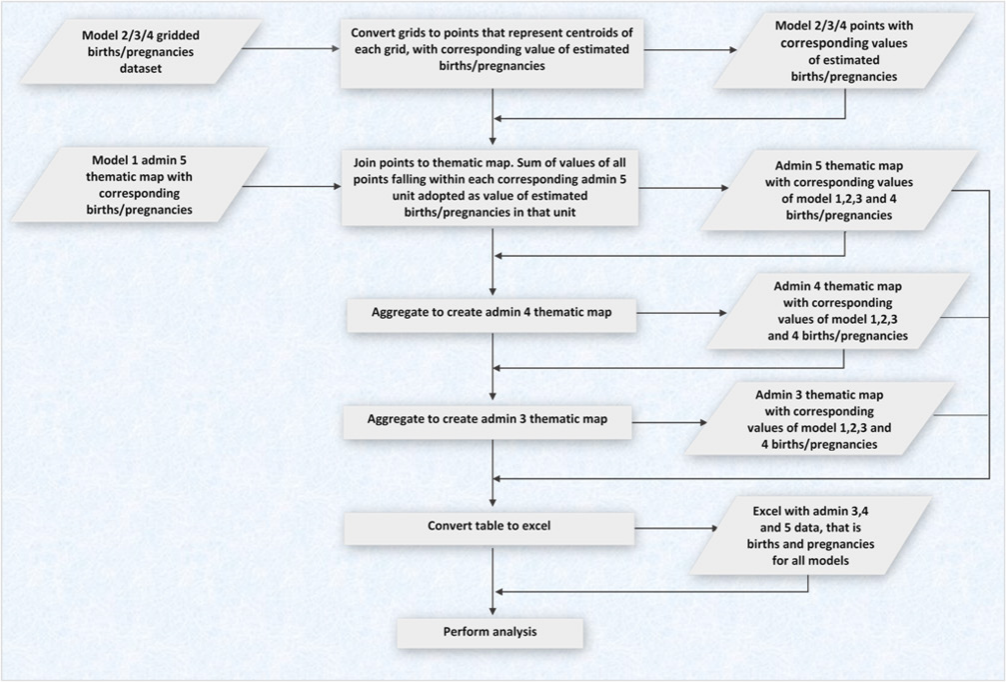
Data preparation process for validation. CLIP, Community Level Intervention for Pre-eclampsia.

To quantify the impact of the model performance on actual births/pregnancy estimates, we converted the Worldpop model outputs to centroid points of the 1 km grids and joined them to admin 5 polygons, by summing the values of the centroid points falling within each polygon, to generate admin 5 polygons with the corresponding values of estimates of live births. This resulted in a thematic map of estimated live births and pregnancies, aggregated to admin 5 level. The CLIP values of births and pregnancies in the excel sheet were also joined to the polygon, resulting in a layer with the following attributes: Name of admin 5-unit, Model 1 (CLIP only) births, Model 1 (CLIP only) pregnancies, Model 2 (Admin 5 Worldpop-CLIP) births. Model 2 (Admin 5 Worldpop-CLIP) pregnancies, Model 3 (Admin 3 Worldpop-CLIP) births, Model 3 (Admin 3 Worldpop-CLIP) pregnancies, Model 4 (Worldpop), births and Model 4 (Worldpop) pregnancies. For these analyses, we compared modelled birth outputs, as pregnancy outputs are dependent on birth estimates. These polygons were dissolved into admin 4 level polygons, creating a map of localities with the corresponding births and pregnancy values of each admin 4 unit for all models. The same was done to create a map of admin 3 units with corresponding values of live births. The process is shown in figure 3.

To compare model prediction errors, we computed the root mean square error (RMSE) across the three administrative unit levels. To enable cross dataset and administrative unit comparison of the prediction errors, the normalised root mean square error (NRMSE) was used. The formulae for both error statistics is shown below:

$$RMSE=\sqrt{\left[n^{-1}\sum_{i=1}^{n}e_{i}^{2}\right]}$$

where $e_{i}$ is the difference between the $i^{th}$ observed (O) and predicted (P) value ($P_{i}$-$O_{i}$) and n is the number of units.

$$NRMSE=\frac{RMSE}{\overset{-}{O}}$$

where $\overset{-}{O}$ is the mean of the observed values.

To determine the impact of input data on model performance, we calculated the difference in NRMSE between model 4 and models 2 and 3. The percentage decrease in prediction error was calculated by dividing the differences by the NRMSE of model 4 at different administrative unit levels and expressing it as a percentage. To quantify the contribution of spatial resolution to the prediction error (expressed as a percentage), the differences in percentage error decrease between models 2 and 3 were averaged. This average percentage value was translated as the proportion of the prediction error due to spatial resolution of input data.

### Ethical considerations

Each head of the household and WRA who participated provided informed consent and this was confirmed by their signature or fingerprint prior to data collection.^33^

## Results

### Average model prediction errors at different administrative unit levels

The model prediction errors are lower at higher administrative unit levels as shown in table 1. All datasets show the same pattern for both the live birth and pregnancy estimates. At all boundary unit levels both model 2 and model 3 have lower model prediction errors than model 4. Models 2 through 4 prediction errors are lowest at the admin 3 level with the livebirths prediction errors of about 0.2, 0.6 and 1.5, respectively and pregnancies prediction errors of about 0.4, 0.3 and 1.2, respectively. The prediction errors for the three models are highest at admin 5 level with the livebirths models’ prediction errors of about 1.1, 1.7 and 2.6, respectively and pregnancies models’ prediction errors of about 0.98, 1.2 and 2.2, respectively. In general, the pregnancies outputs of the three models have lower prediction error than the births datasets at all administrative unit levels, except for Model 2 (Admin 5 Worldpop-CLIP) at both admin 4 and admin 3 levels, where the live births model has lower prediction error than the pregnancies model.

**Table 1:** NRMSE prediction errors of different administrative unit levels

Administrative level	Model 2		Model 3		Model 4	
	Births	Pregnancies	Births	Pregnancies	Births	Pregnancies
Admin 5	1.1463	0.9784	1.6749	1.2260	2.5590	2.2172
Admin 4	0.3472	0.4617	0.7531	0.4453	1.5986	1.3578
Admin 3	0.2056	0.3651	0.5625	0.2758	1.4889	1.2407

NRMSE, normalised root mean square error.

### Effect of accuracy and spatial resolution of input data on live births dataset

Table 2 shows that, using CLIP data at admin 3 level reduces the prediction error of the model by at least 34.5% at admin 5 level and 62.2% at admin 3 level. Using the same input data at a higher spatial resolution, that is at admin 5 level, reduces the prediction error of model 4 (Worldpop) by at least 55.2% at admin 5 level and 86.2% at admin 3 level. In general, increasing the spatial resolution of the input data from admin 3 to admin 5 units reduces the prediction error of the model by an average 23.3%.

**Table 2:** Percentage change in NRMSE between models

Administrative level	Model 2	Model 3	Model 4	% Error decrease		% Error due to spatial resolution
				Admin 3 data	Admin 5 data	
Admin 5	1.1463	1.6749	2.5590	34.55	55.20	20.65
Admin 4	1.5986	0.7531	0.3472	52.89	78.28	25.39
Admin 3	1.4888	0.5625	0.2056	62.22	86.19	23.97

NRMSE, normalised root mean square error.

**Figure 4:**
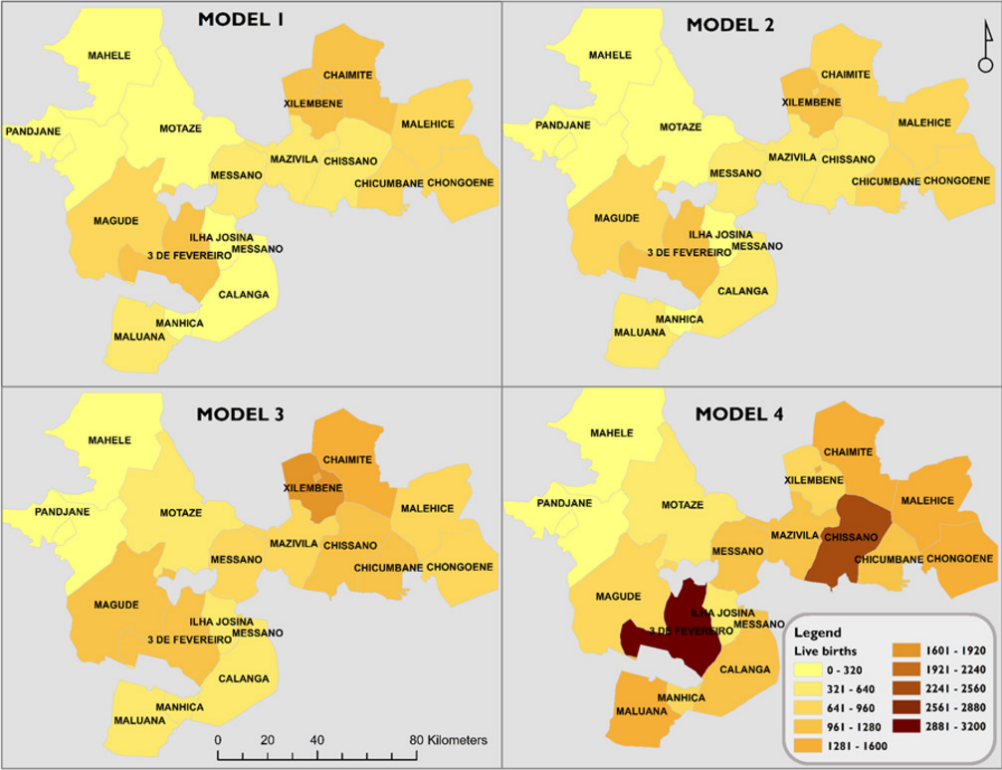
Aggregated live births at admin 3 level.

**Figure 5:**
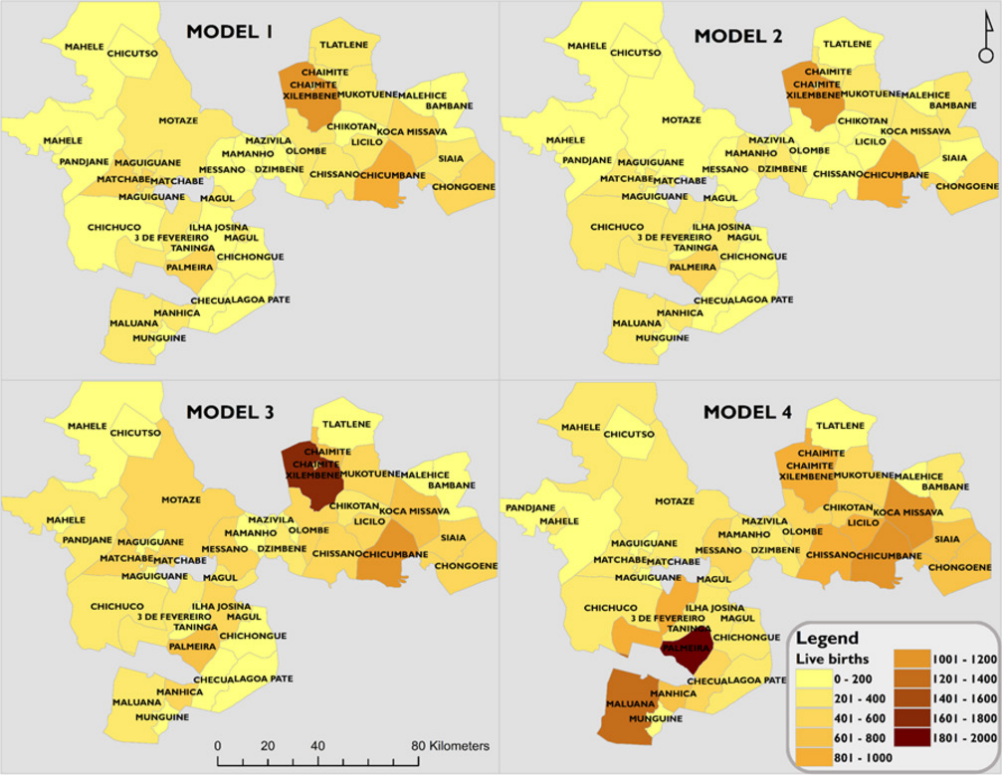
Aggregated live births at admin 4 level.

The thematic maps show model outputs for models 1 through 4, with live births at both admin 3 level (figure 4) and admin 4 (figure 5). The thematic map created through model 2 (Admin 5 Worldpop-CLIP) is the one most like the map created using CLIP data in terms of the range of values. Concerning the representation of relative values within the maps, model 3 (Admin 3 Worldpop-CLIP) performs better at admin 4 level in representing the relative values shown in the map as compared with model 1 (CLIP only) values.

**Figure 6:**
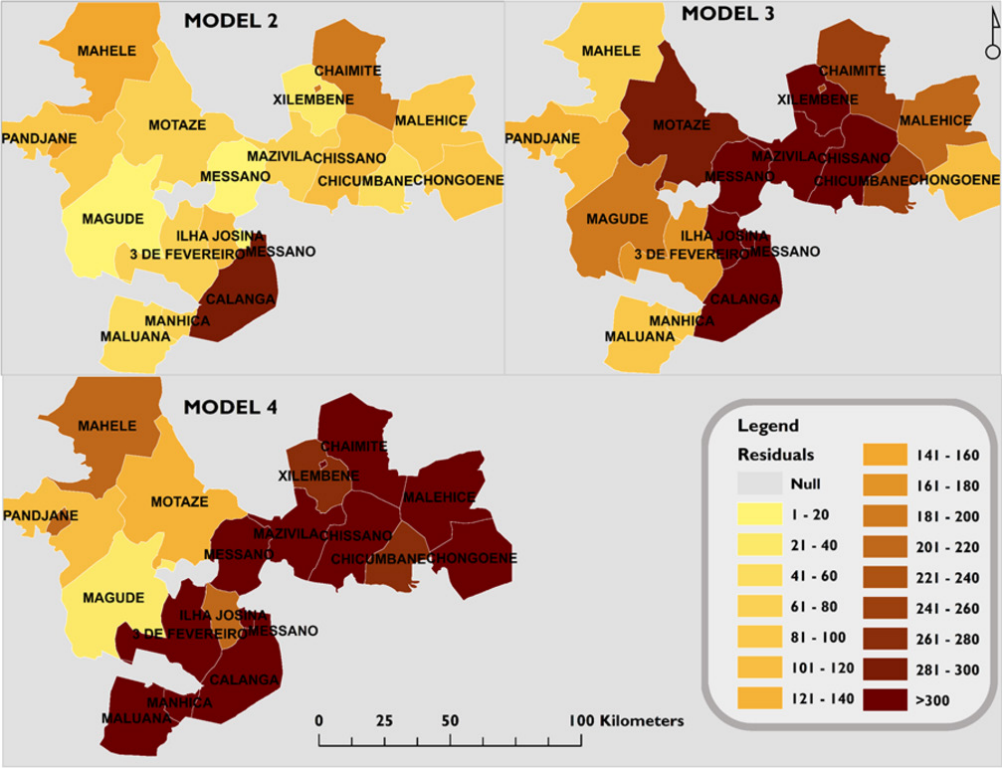
Admin 3 level maps showing the residuals obtained from difference in estimated births between model 1 (CLIP only) and the other models. CLIP, Community Level Intervention for Pre-eclampsia.

**Figure 7:**
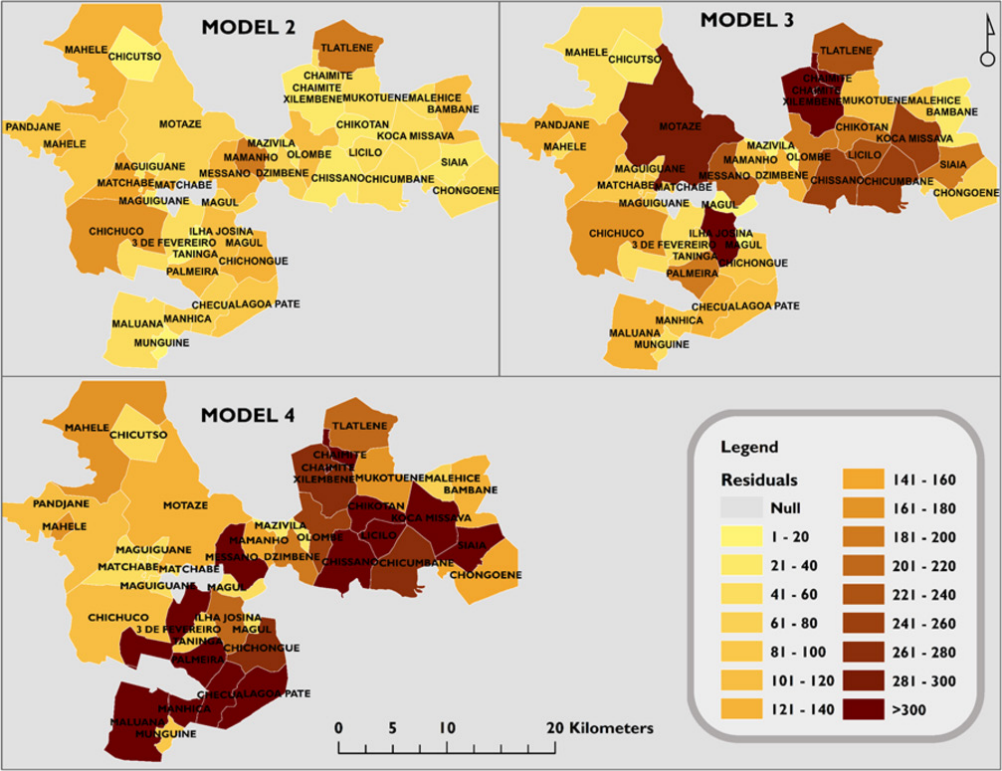
Admin 4 level maps showing the residuals obtained from difference in estimated births between model 1 (CLIP only) and the other models. CLIP, Community Level Intervention for Pre-eclampsia.

Figure 6 and figure 7 show the distribution of residuals at admin 3 and 4 levels, respectively. The residuals were obtained by calculating the difference between model 1 values of live births and the other three models. The darkest regions represent regions with residuals greater than 300 births. As seen in the maps, model 2 better estimates the CLIP births (represented by model 1) compared with the other models at both admin 3 and 4 levels.

## Discussion

The results showed that this model performs well in estimating live births and pregnancies at the highest level of spatial resolution, especially with improved spatial and temporal resolution of the input data. However, benchmarking these model approaches on a diverse set of areas, with sufficient high-quality knowledge base will provide sufficient evidence on how well the models perform. A huge amount of health data in LMICs is filed and not effectively used for analyses that can influence decision making due to its decentralisation, making it difficult for researchers to consolidate the data for analyses.^37^ The validation of model outputs has been mainly relative, with the focus being model comparison of performance, rather than comparison of outputs.^38^ Spatial scale of validation also differs from one author to another, meaning different authors only validated performance of the datasets at one spatial scale and did not explore the changes of prediction errors from one spatial scale to the other.^21 39–41^ It is essential to validate performance of a model at different spatial scales because different disaggregation methods are affected by spatial scale of available census data.^38^ Such findings have not been explored in several studies. In this study, this difference was explored by comparing the prediction errors of all the models at different administrative unit levels.

This study focused on model comparison with varying input data, using methods established through the Worldpop project, using novel, region-specific data enumerating actual births and pregnancies. Here, we quantified the role of input census data, examining model performance for varying input spatial resolutions. The impact of the model error is shown by the prediction errors of the pregnancy and live birth datasets at different administrative unit levels. This error has been shown to have less impact on the accuracy of the datasets at higher administrative levels. We found that the spatial resolution of input data had a significant effect on model 4’s prediction accuracy of the live birth and pregnancy values.

Recent studies have been focusing on methods for improving delineation of urban, suburban and rural areas. These methods are essential in the definition and demarcation of urban, suburban and rural boundaries, which improve the accuracy of estimates that are modelled using the rural/urban classification.^35^ Methods involving the use of satellite imagery data have proven effective in classifying settlement types. The use of spectral reflectance and night-time lights data obtained from satellite imagery are methods that are effective in delineating settlement types.^42^ Night-time imagery data are also effective in modelling health indicators (like crude birth rates) at subnational levels, making it useful when predicting such health metrics, as a strong correlation between health and development (like level of electrification and district domestic product) has been shown to exist.^43^

Despite their limitation of being expensive, use of remotely sensed data like spectral and/or textural metrics or demographic information and distance-to-services metrics at higher and more detailed resolutions, increases their potential of better performance in producing datasets with better accuracy.^44^ The use of high resolution ortho-rectified RapidEye archive data for settlement has a high potential of being replicated for the other countries to allow improvement especially in the detail of the dataset.^38^ Integration of geotweets data into the methods used in the production of the demographic datasets proved to improve the accuracy and level of detail of the datasets.^21^ The strength of its application, however, is in the density of geotweets in the whole region, that is the higher the density of active twitter users the greater the potential of the use of this method.

Use of mobile phone geolocation data to disaggregate census data has been proven to improve the accuracy of population densities as it captures the dynamic nature of populations^45 46^ while predicting inter-census period population using models trained on known census data.^41^ However, like geo-located tweets, its accuracy is directly dependent on the network structure, thus the higher the density of the towers, the higher the precision of the mobile phone communication geo-location.^45^ Although remote sensing methods produce predictions with a higher precision but less accuracy, with an overestimation of population densities in low-density areas and an underestimation of population densities in high-density areas.^45^

### Limitations

The CLIP project mapped only households with WRA and although insignificant, the number of pregnant women below the age of 15 and above the age of 49 were also recorded.^33^ The models however were created using only the data for the ages 15–49 and the population dataset that represented populated areas and not just the areas with WRA. The analysis to determine how accurately the population model identified populated areas at grid cell level was therefore not done. It is important to note that these results only apply in the regions of Southern Mozambique, a very small fraction of the whole dataset. It is not reflective of the entire dataset. Regions with a different geography from that of southern Mozambique may yield different performance results. The study area, which is the rural regions of southern Mozambique, does not provide a holistic picture about how the models perform at different settlement settings, that is urban, suburban and rural settings. Performing the analyses in regions with diverse settlement settings using high resolution data with comprehensive coverage will provide evidence on how well the models detect changes from one settlement setting to the next. The RMSEs were computed with the assumption that the weight of all the residuals is 1 instead of assigning different weights.^47^ However, it is known that accuracy of disaggregation is also dependent on the non-intuitive relationships between population density and the supporting covariates of the areas being mapped.^48^

Satellites have been the most commonly used source of ancillary data in the form of land cover and land use data used for estimation of population densities because of the high correlation between land use/land cover (LULC) category and population density.^20 49 50^ Some remotely sensed data sources used for large scale demographic maps, however, have resolutions that are too low for obtaining accurate disaggregated data especially for urban areas which are highly heterogeneous.^44^ The limitation of using remotely sensed data (whether high or low resolution) is that it cannot be reliably derived by any known algorithm due to the assignment of weights to the LULC classes being based on heuristic rules and assumptions without a solid evidence base for such rules.^20 51^ Another limitation of using land cover data, especially in heterogeneous urban areas, is the overestimation of population densities in certain land cover classes like ‘developed, open space’, due to the category being intermingled with other urban categories having high population density.^49^ Such factors are to be taken into consideration for weighting when computing the prediction error of a demographic distribution dataset.

## Conclusion

There is need for more studies that will compare the global datasets against independent demographic datasets for individual countries. Previous methods used have focused more on comparing population distributions. Most studies have demonstrated the desire to create datasets independent of boundary data as boundary data require good documentation and accuracy to produce quality datasets.^13^ Lack of such data especially in the developing countries presents problems in mapping hence eagerness of the authors to explore more and more methods that do not require boundary data.^52^

There is need for more data collection techniques that conduct comprehensive censuses like the CLIP project. It is also imperative for such projects to take advantage of the power of mapping tools at their disposal to fill the gaps in availability of datasets for populated areas. This is made possible by, for example, mapping all the households despite not inhabiting populations with the variables of interest. With the technologies that allow data sharing, health research data collected now have expanded their applications in multiple disciplines, hence it is of great importance to always consider such potential when collecting health data.

The global data sets’ potential of producing high quality data is great. Different studies have shown that more and more methods are being unveiled, with the advent of technologies that allow location of populations in real time, that will improve these datasets, providing free access to high quality demographic distribution data. Availability of such data on demand will enormously improve performance of intervention programmes by reducing the amount of resources used in accumulating data from different sources to perform analyses.
